# A Rare Case of Acute Idiopathic Pancreatitis in Third Trimester Which Aggravated in Early Postpartum Period

**DOI:** 10.7759/cureus.7348

**Published:** 2020-03-21

**Authors:** Washma Amir, Marrium Nawaz, Zohaib Ahmed

**Affiliations:** 1 Internal Medicine, Dow University of Health Sciences, Karachi, PAK

**Keywords:** acute pancreatitis, idiopathic pancreatitis, pregnancy, postpartum, third trimester, idiopathic pancreatitis in pregnancy

## Abstract

Acute pancreatitis (AP) in pregnancy and post-partum period is a rare event and can have a lethal effect on the mother and the fetus. Gallstone disease is thought to be the most common causative factor of AP; however, in many cases the cause remains unclear. Here, we present a case of severe AP occurring in late pregnancy which aggravated in the early postpartum period. A 32-year-old multiparous woman, para 7, presented with severe abdominal pain, abdominal distension and multiple episodes of vomiting. The pain was localized to the upper abdomen and radiating to the back, aggravated by food and bending forward. She had neither a history of chronic alcoholism nor any evidence of viral infection was found. The patient was diagnosed with idiopathic severe AP on contrast-enhanced computed tomography, which was managed conservatively and recovered within several days. She did not have any recurrence thereafter and had a good clinical recovery. Therefore, it is important to consider AP when a woman presents with upper abdominal pain, nausea and vomiting in pregnancy and during the postpartum period to improve the maternal outcome for patients with AP.

## Introduction

Acute pancreatitis (AP) is a common problem with an incidence of five to 80 per 100,000 of the general population annually [1]. However, in pregnancy, it occurs infrequently and has a reported incidence of approximately one in 1,000 to three in 10,000 births. It is rare during the first and second trimesters of pregnancy (12%), it usually occurs during the third trimester (52%) and 30% occurs in the postpartum period [1,2]. During pregnancy, AP’s etiological associations are similar to those in the general population. The most common identified causes of AP are gallstones, alcohol abuse, familial hyper-triglyceridemic-induced pancreatitis and idiopathic pancreatitis [1-4]. While its onset is acute, the spectrum of AP in pregnancy ranges from mild pancreatitis to complications like necrosis, abscesses, pseudocysts and multiple organ dysfunction syndromes. Older reviews of AP in pregnancy reported maternal and fetal mortality rates as high as 20% and 50%, respectively, but latest advancements in diagnostic technology have enabled early diagnosis and management which have played an important role in improving the prognosis [1,2]. Herein, we report a rare misdiagnosed case of idiopathic AP in late pregnancy which aggravated in the postpartum period and discuss its management.

## Case presentation

A previously healthy 32-year-old multiparous woman, gravida 7, para 6 with no known comorbids, developed complaints of hyperemesis and mild abdominal pain at 36th week of gestation. The condition was misdiagnosed as an effect of pregnancy and was treated symptomatically at a tertiary care hospital. Later, she delivered a viable healthy male infant by normal spontaneous vaginal delivery at 39 weeks of the pregnancy.

After 10 days of delivery, she was admitted due to severe abdominal pain with distension and multiple episodes of vomiting, leading us to suspect acute hepatitis, acute cholecystitis and AP as differentials. The pain was localized to the upper abdomen and radiating to the back, aggravated by food and bending forward. She had no relevant past medical history or family history of autoimmune disease.

On examination, the abdomen was distended and slightly tender; fluid thrill and shifting dullness were positive with decreased bowel sounds. On respiratory examination, bilateral chest expansion was also decreased accompanied with dull vocal resonance and on percussion, a stony dull note was heard in the lower third of the left lung. Over the next day, the patient started to develop respiratory distress for which she was shifted to a high dependency unit (HDU). She was conscious and oriented on admission to HDU.

Her vital monitoring showed a pulse rate 100/min, blood pressure 120/70 mmHg, the body temperature 102°F and a respiratory rate 30/min. Arterial blood gas was pH 7.32, PaO_2_ 80 mmHg, PaCO 242.5 mmHg and HCO_3_ 22.1. Laboratory investigations revealed Hb 9.6 g/dL, TLC 10,600/mm^3^, platelet 417,000/mm^3^, C-reactive protein 43.7, blood urea nitrogen 9 mg/dL, creatinine 0.5 mg/dL, serum amylase 418U/L, lipase 178 U/L and triglyceride 128 mg/dL. Viral markers, hepatitis B surface antigen (HBsAg) and hepatitis C antibody (anti-HCV), were also tested which came out negative.

**Figure 1:**
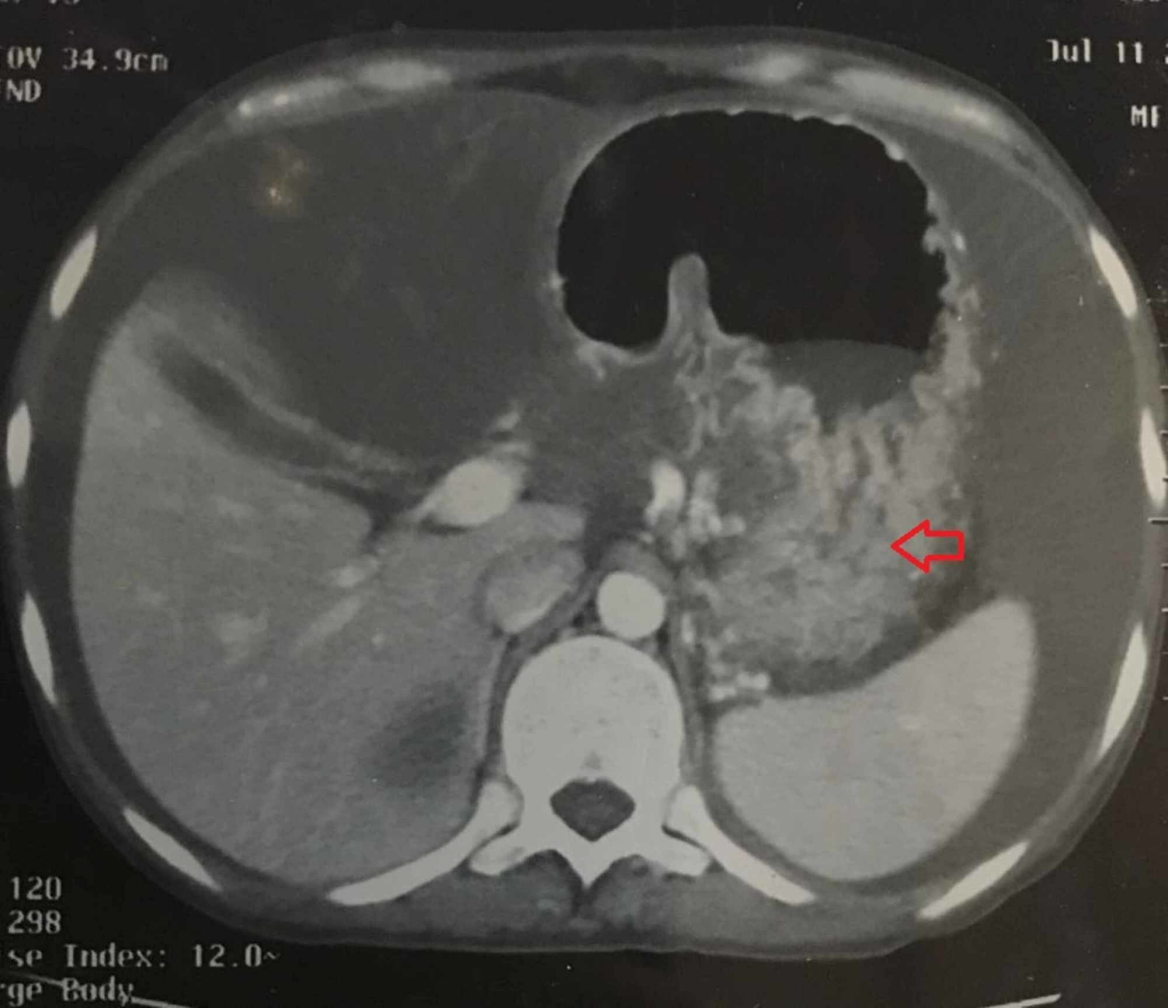
Abdominal CT scan showing cystic collection

**Figure 2:**
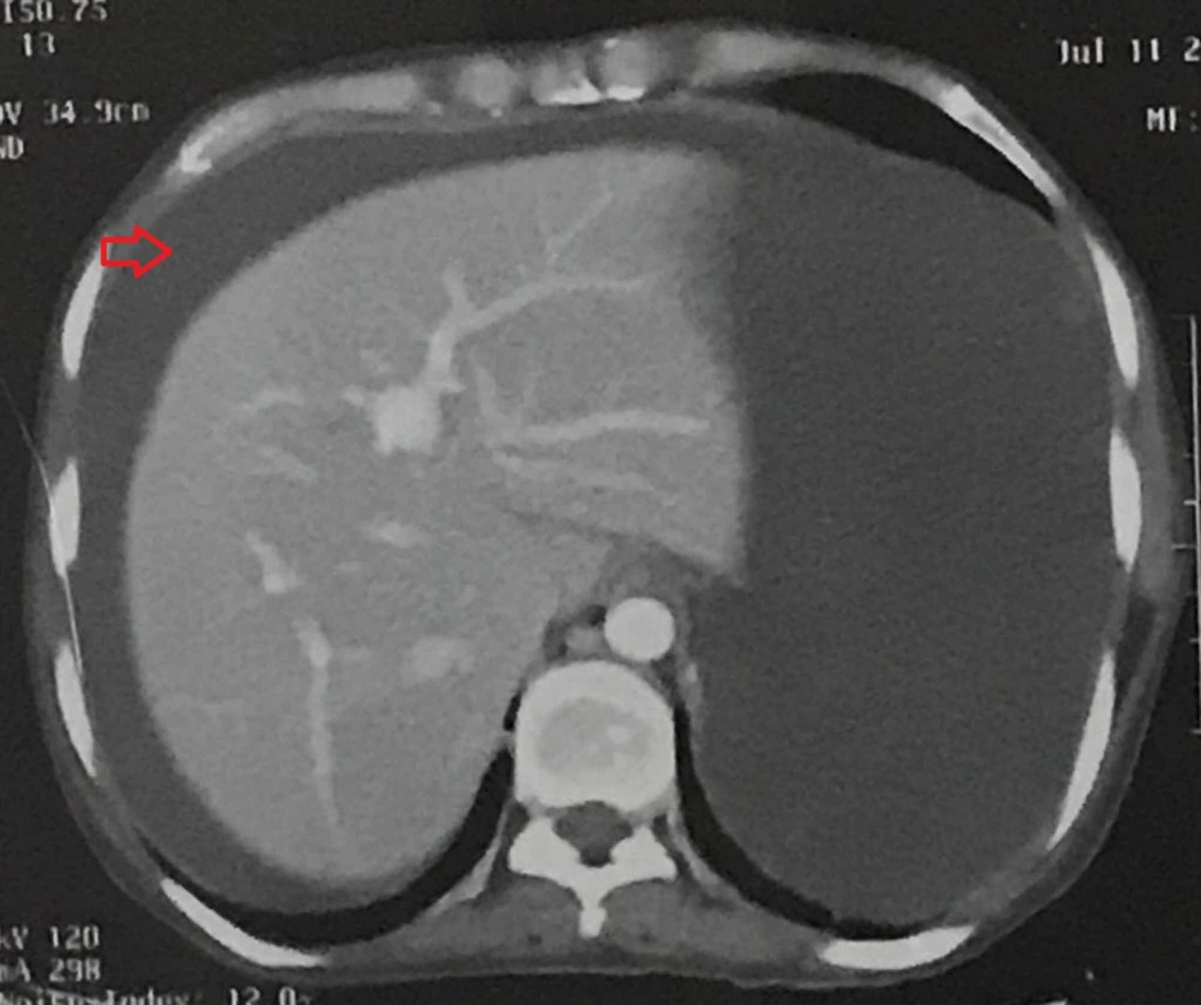
CT scan of the abdomen showing significant gross ascites

In further workup, contrast-enhanced computed tomography (CECT) of the abdomen revealed a walled-off, peripherally enhancing cystic collection identified in the anterior peripancreatic region, which appeared to be communicating with the pancreatic duct along with multiple small pockets of collection in the peripancreatic space (Figure 1), with significant gross ascites (Figure 2) and smooth peritoneal thickening.

There was no evidence of gallstones or biliary sludge. Although the cause remained unclear, she was diagnosed as a case of idiopathic AP. Pleural effusion, which caused the worsening of respiratory symptoms, developed secondary to severe AP. To resolve this, a pleural chest tube was placed with drain for six days. The patient was managed conservatively with intravenous fluids, broad-spectrum antibiotics, analgesics and nil per oral.

The clinical condition of the patient improved gradually and was shifted to the ward for further management. She was hemodynamically stable, afebrile, breathing normally and was advised soft diets. Serum amylase and lipase levels dropped to 96 and 62 IU/L, respectively. She was discharged from the ward after 19 days and was found to have excellent clinical recovery in her follow-up visit after one month.

## Discussion

AP seldom complicates pregnancy, with an occurrence of about one in 1,000 to three in 10,000 births [1,2]. The occurrence of AP rises with gestational age; over half (52%) occurs in the third trimester, while it is rare during the first and second trimesters of pregnancy (12%) and only 30% cases occur in the postpartum period [1,2,4]. Out of the 43 cases of AP in pregnancy reported by Ramin et al., only one case occurred in the puerperium [5]. In our case, AP occurred in the third trimester which aggravated in the early postpartum period. 

The pathogenesis of AP in pregnancy is similar to that in the general population but due to rare incidence, the relationship between pregnancy and pancreatitis remains unclear. According to several studies, gallstones are the most common cause of AP during pregnancy responsible for more than 70% of cases [1-7]. The association of AP with gallstones in pregnancy and puerperium is most likely due to the known alterations in bile composition, gallbladder contractility and sludge formation that occur during and after pregnancy [1-3]. Weight gain and hormonal changes also predispose pregnant women to biliary sludge and gallstone formation [2]. Estrogen increases cholesterol secretion in hepatic bile leading to its supersaturation, while progesterone in pregnancy causes an increase in gallbladder volume and slows emptying which induces bile stasis and eventually gallstone formation in late pregnancy [1-3]. In the third trimester, increased intra-abdominal pressure on the biliary duct and an enlarged uterus increase the risk of AP in late pregnancy and early puerperium [1,3].

Hyperlipidemia is the second most common causative factor of AP. Triglycerides levels gradually rise 2.5-fold over prepregnancy levels, reaching its peak during the third trimester due to decreased activity of enzyme lipoprotein lipase, and then decrease gradually during postpartum over a span of six weeks, predisposing women to increased risk of AP more often during the third trimester and postpartum [1-4].

Other etiological factors for AP include alcohol abuse (10% of cases), familial hypertriglyceridemic-induced pancreatitis (5%) and idiopathic pancreatitis (15%). Rarely, hyperparathyroidism, connective tissue diseases, abdominal surgery, infections or iatrogenic sources such as diuretics, antibiotics and antihypertensive drugs are reported [1-4,6,8,9].

The clinical presentation of AP during pregnancy is similar to that of non-pregnant patients: symptoms inlcude mild to moderate epigastric pain, nausea, vomiting, abdominal distention, low-grade fever, tachycardia, hypotension and abdominal tenderness. Rarely patients might have systemic inflammatory response syndrome causing acute respiratory distress syndrome [1,3,4,9]. Although several other disease states can have a similar presentation as pancreatitis like duodenal ulcer perforation, cholecystitis, hepatitis, bowel obstruction, HELLP (hemolysis, elevated liver enzymes, low platelet count) syndrome and pre-eclampsia, it is necessary to distinguish between these before appropriate treatment can begin.

The diagnosis of AP can be difficult as hematological and biochemical modifications related to pregnancy can influence the interpretation of tests and the assessment of its severity. Laboratory tests are essential for diagnosing AP and include serum amylase, lipase, complete blood count, serum triglycerides, calcium and liver function tests. An elevated serum amylase and/or lipase level greater than three times average value has good predictive value for diagnosing AP in pregnant women. In our patient, the amylase level was raised to 418 U/L and lipase level to 178 U/L. To rule out AP due to hypertriglyceridemia, its levels were tested which came out to be within the normal range. Since hepatitis B and C can be the cause of AP, viral markers were also investigated, which came out to be negative in our patient. CECT provides the best imaging of the pancreas and surrounding structures. It is particularly helpful in assessing complications [1,2,9]. In our case, CT showed peripancreatic inflammation, distorted pancreatic contour and gross ascites.

Considering these investigations and no history of alcohol intake, the cause of her AP remained unclear; thus, we diagnosed her with idiopathic AP. It is reported that 15% of patients with AP had idiopathic disease [1-3,9].

Management of AP in the pregnant patient is often guided by the management of AP in non-pregnant patients. AP is usually self-limited, and the majority of patients respond to initial conservative measures which include intravenous hydration, nil per oral, analgesics, gastric decompression, antispasmodic drugs and antibiotics [1,2,9]. The surgical approach remains controversial [1]. Most cases of AP resolve spontaneously and in 90% of cases, inflammation subsides within one week [7,9]. However, about 10% of patients may develop serious complications which might manifest as necrosis and multiple organ failure, including cardiovascular, pulmonary and renal systems. Severe respiratory distress due to pleural effusion developed in our patient and had to be managed in an intensive care unit. Pancreatic pseudocysts complicate 5% of the cases of pancreatitis and can be detected on CT [2,9]. Managing this rare condition is not systematic, and as these have spontaneous resolution rate of 30%-40%, observation is the recommended approach for asymptomatic patients [2,10]. Our patient developed pseudocysts which resolved spontaneously during her hospital stay.

Although it is a rare condition, AP must be considered when evaluating women presenting with abdominal pain in pregnancy and postpartum. Appropriate lab investigations, specifically amylase and lipase levels, must be done. Radiological imaging is necessary to evaluate the etiology of raised amylase and lipase levels. It may serve to boost the effect of early detection and prompt intervention for its management.

## Conclusions

We have discussed a case of idiopathic AP which developed in the third trimester and worsened in the early postpartum. Due to its similarity with other diseases, differentiating the disorder can be puzzling. Although uncommon, it should be considered as a differential when a woman presents with acute abdomen in pregnancy or postpartum. Early diagnosis and classification of severity of AP at presentation are an essential step for successful management because delayed recognition and treatment can allow for the greater prevalence of associated complications.
